# Comparing Three Dual-Task Methods and the Relationship to Physical and Cognitive Impairment in People with Multiple Sclerosis and Controls

**DOI:** 10.1155/2015/650645

**Published:** 2015-11-23

**Authors:** Megan C. Kirkland, Elizabeth M. Wallack, Samantha N. Rancourt, Michelle Ploughman

**Affiliations:** Recovery & Performance Laboratory, L.A. Miller Centre, Faculty of Medicine, Memorial University, 100 Forest Road, St. John's, NL, Canada A1A 1E5

## Abstract

Dual-tasking (DT) is a measure to detect impairments in people with multiple sclerosis (MS). We compared three DT methods to determine whether cognitive (Montreal Cognitive Assessment (MoCA)) or physical disability (Expanded Disease Severity Scale; EDSS) was related to DT performance. We recruited MS participants with low disability (<3 EDSS, *n* = 13) and high disability (≥3 EDSS, *n* = 9) and matched controls (*n* = 13). Participants walked at self-selected (SS) speed on an instrumented walkway (Protokinetics, Havertown, USA), followed by DT walks in randomized order: DT ABC (reciting every second letter of the alphabet), DT 7 (serially subtracting 7's from 100), and DT 3 (counting upwards, leaving out multiples and numbers that include 3). DT 7 resulted in the most consistent changes in performance. Both MS and control groups reduced velocity and cadence and shortened step length during DT with no significant differences between groups. Control subjects widened stride width by about 1 cm while MS subjects (collapsed as one group) did not. MS subjects with higher disability significantly increased percentage time in double support during DT compared to SS (*F* = 12.95, *p* < 0.001). The change in DS was related to cognitive and not physical disability (*r* = 0.54,  *p* < 0.05).

## 1. Introduction

Multiple sclerosis (MS) is an autoimmune neurodegenerative disease, usually diagnosed between the ages of 20 and 40 [1]. People with MS (PwMS) experience unpredictable symptoms such as weakness, fatigue, and cognitive impairment [2–5].

PwMS often describe feeling unsteady while walking which is worsened when combined with another task such as talking [6]. Challenges when completing two tasks at once, also called dual-tasking or cognitive interference, divide attention and can cause falls among people with central nervous system impairments [7, 8]. The ability to dual-task (DT) is emerging as a potential outcome in rehabilitation and in MS [9]. Although authors report that adding a cognitive task to walking slows gait velocity in PwMS [10], it is not known whether the impairment is peculiar to PwMS (compared to controls) or whether gait velocity is the most important gait parameter to evaluate [7, 11–13]. In one review of DT testing in PwMS [7], five of the fourteen studies compared MS subjects to controls [6, 14–17] and only two of these matched for education, age, and gender [14, 17]. Considering that level of education could impact cognitive performance, it would be reasonable to match education level when recruiting control subjects. Furthermore, it is not clear which method of cognitive interference best detects impairments during DT testing [7]. A frequently used method requires the subject to serially subtract sevens beginning with 100 while walking [8, 18, 19]; however the technique has been criticized because it was considered frustrating for subjects [18]. The relationship of DT to disability is not clear [14, 16]. Some authors report that changes in gait during DT correlated with cognitive and physical impairment [11] while others suggest it is related to physical variables only [20].

We aimed to measure the impact of DT on gait parameters in MS subjects with low and higher disability levels compared to controls and determine which of three DT methods consistently affected gait. We also aimed to determine whether cognitive or physical status was related to DT performance.

## 2. Methods

### 2.1. Participants

Following approval by the Health Research Ethics Authority, participants with a neurologist-confirmed diagnosis of MS according to the 2010 McDonald criteria were recruited from the MS clinic or the rehabilitation hospital outpatient service [21]. They were included if they were older than 18 years of age, relapse-free in the previous three months, and able to provide consent. They were excluded if they had musculoskeletal impediments to walking. The most recent Expanded Disease Severity Scale (EDSS) score was extracted from the health record. Participants with EDSS < 3 were assigned to the low disability group and those scoring EDSS ≥ 3 to the high disability group. To create a comparison group, we recruited a convenience sample of age (±3 years), gender, and education (±3 years) matched subjects without MS or any musculoskeletal or neurological conditions.

We calculated sample size based on mean and SD of two previous studies examining DT in subjects with MS and healthy controls [15, 22]. Velocity at self-selected (SS) walking was used for the calculation [15, 22]. With significance set at *p* < 0.05 and power at 0.8 and depending on which study was considered, the sample size was estimated at 6 or 12 per group. We aimed to recruit 10 subjects in each group (low disability, high disability, and controls).

### 2.2. Protocol

Subjects attended one testing session. After obtaining consent and collecting demographic information, participants completed the Montreal Cognitive Assessment (MoCA; scored out of 30) which has been tested in MS patients [23]. Participants walked at their comfortable walking speed (SS) twice along an instrumented walkway (1.2 × 4.3 m, PKMAS, Protokinetics Inc., Havertown, PA). They then completed three DT conditions in random order with directions provided using a standard script, walking twice across the walkway during each method. The three DT methods included reciting alternate letters of the alphabet beginning with B (DT ABC), counting backwards from 100 by subtracting 7's (DT 7), or reciting numbers beginning with the number 1 while excluding those which included the digit 3 or numbers which were multiples of 3 (DT 3). DT ABC had been previously tested in MS [10] and DT 7 in Alzheimer's disease [18]. The DT 3 was a new method devised to be a simple nonrhythmical task.

### 2.3. Gait Variables

Forces and location of forces using an* x-y-z* coordinate system were calculated by the walkway software. Variables included average stride length, average stride width, average percentage of time in double support, average velocity, and average cadence [10, 11, 14]. Stride length (cm) was the distance from the heel of one foot to the subsequent heel strike of the same foot. Stride width (cm) was the distance between a line connecting the two ipsilateral foot heel contacts and the contralateral foot heel contact perpendicular to the stride. Percentage time in double support (DS) was the sum of all periods of time when both feet were in contact with the ground, as percentage of total gait cycle time. Velocity was calculated by dividing the sum of all the stride length measurements by the sum of all stride time measurements, presented in cm/s. Lastly, cadence (steps/min) was the total number of footfalls minus one, dividing by the ambulation time and multiplying the result by 60. Since we expected that subjects could also exhibit gait variability during DT, we also extracted the coefficient of variability (CV) for each variable listed above.

### 2.4. Data Analysis

After removing incomplete footfalls, data was exported and analyzed in SPSSv21. We calculated dual-task cost (DTC) using the previously published equation, where SS was self-selected pace output and DT was a dual-task output for a specific variable [10–12, 15]:

(1) $$\begin{array}{c} \text{DTC}=\frac{SS-DT}{SS}\times 100\%. \end{array}$$

We used descriptive statistics and *t*-tests to compare demographic characteristics between groups and for the categorical variables chi-square. Effect of group (MS subjects or controls), condition (SS and DT conditions), and group × condition interaction was compared using two-way repeated measures ANOVA. Significance was set at *p* < 0.05 and if there was a significant effect of group or condition, post hoc comparisons were completed (Bonferroni). We examined the correlations between DTC and the MoCA score (cognition) or EDSS (MS-related disability) using bivariate correlations (Pearson coefficient, with significance set to *p* < 0.05).

## 3. Results

### 3.1. Participants

We recruited 11 MS participants with low disability (<3 EDSS; 7 females, 4 males), 9 MS participants with high disability (≥3 EDSS; 6 females, 3 males), and 13 controls (8 females, 5 males). Of the participants with MS, 15 were diagnosed with relapsing-remitting (RRMS) and five as secondary progressive MS. Some control subjects matched more than one participant with MS. There were no differences in age (*F* = 0.76, *p* = 0.48), gender (*F* = 0.03, *p* = 0.97), or education (*F* = 0.11, *p* = 0.90) between groups (Table 1). Since the order of the dual-task conditions was randomly assigned, we tested to determine if there was an effect of order on all the reported variables. Of the five gait variables and four conditions (SS and 3 DT conditions), only velocity during DT 7 task was affected by order (*F* = 2.64, *p* = 0.05). We applied a more stringent significance value of *p* < 0.01 to account for this effect during analysis of velocity.

### 3.2. Effects of DT in MS Participants and Controls

As expected, for stride length, velocity, and cadence, there was a significant decrement in performance in both MS subjects and controls during DT (Table 2). All groups exhibited similar shortening of stride length by about 11–19 cm with significant effect of condition (*F* = 20.28, *p* < 0.0001) but no effect of group (*F* = 1.55, *p* = 0.23) or group × condition interaction (*F* = 1.13, *p* = 0.35). Post hoc analysis showed that all DT methods in the MS groups resulted in reduced stride length compared to SS. In the control group, only DT 7 resulted in decreased stride length.

With the addition of DT, walking velocity decreased by about 21–46 cm/s with a significant effect of condition (*F* = 42.04, *p* < 0.0001) but no effect of group (*F* = 1.05, *p* = 0.36) or group × condition (*F* = 1.90, *p* = 0.09). Post hoc analysis showed that all subject groups exhibited reduced velocity during DT compared to SS with no difference between the DT methods. Similar to velocity effects, cadence also decreased by about 13–31 steps/min during DT compared to SS with a significant effect of condition (*F* = 27.93, *p* < 0.0001) but no effect of group (*F* = 1.04, *p* = 0.37) or group × condition (*F* = 2.88, *p* = 0.07).

Two variables, DS and stride width, were important in distinguishing between subject groups. With respect to DS, we were able to distinguish the MS participants with higher disability from controls. Repeated measures ANOVA revealed a significant effect of condition (*F* = 13.99, *p* < 0.0001) and group (*F* = 4.14, *p* = 0.03) but not group × condition (*F* = 0.60, *p* = 0.73). As expected, at SS speed, the high disability group spent significantly longer time in DS (35.1% SD9.8) compared to the low disability group (28.6% SD2.8) and controls (27.3% SD2.6). MS subjects in high and low disability groups increased time in DS during DT compared to SS, adding about an additional 4% (*F* = 11.72, *p* < 0.0001; *F* = 5.36, *p* < 0.01), while control subjects did not (*F* = 2.45, *p* = 0.08). In terms of stride width, there was no significant effect of condition (*F* = 1.88, *p* = 0.14), group (*F* = 2.26, *p* = 0.12), or condition × group (*F* = 1.31, *p* = 0.26). However, when comparing the combined MS groups with controls, there was an effect of condition approaching significance (*F* = 2.61, *p* = 0.06) and no effect of group (*F* = 0.00, *p* = 0.98) and condition × group (*F* = 2.43, *p* = 0.07). Control subjects widened their stride width on average 1 cm with the addition of a DT whereas MS subjects did not demonstrate this behavior (Table 2). In fact, in some DT conditions there was a trend, although not significant, of narrowed stride width in MS subjects.

We calculated CV for each gait variable (stride length, stride width, DS, and stride time, resp.). There was no effect of group, condition, or group × condition interaction (data not shown).

### 3.3. Comparing Methods to Detect the Impact of DT

We combined the low and high disability MS groups to compare DTC across the three DT methods (Table 3). DT 7 condition produced the largest and most consistent DTC in stride length and DS in the MS group. In terms of stride length and DS, there was an effect of condition (*F* = 4.81, *p* = 0.01; *F* = 4.08, *p* = 0.02), but not group (*F* = 1.81, *p* = 0.18; *F* = 0.69, *p* = 0.51) or group × condition (*F* = 0.33, *p* = 0.86; *F* = 0.75, *p* = 0.56). There was no significant effect of group, condition, or group × condition in DTC of gait variables: velocity, cadence, or CV (data not shown). When examining stride width, although there was no significance of group (*F* = 3.13, *p* = 0.06), condition (*F* = 1.46, *p* = 0.24), or group × condition (*F* = 0.65, *p* = 0.63), the group effect approached significance. Post hoc analysis showed that the DTC of stride width in MS subjects was less than controls (*p* < 0.05; all DT methods; Table 3).

### 3.4. Relationship between DT Performance and Cognitive and Physical Disability

Firstly, there was no correlation between cognitive score measured using MoCA and any DTC in control subjects (*p* > 0.05; data not shown). In MS subjects, cognitive score was not related to gait variables at SS speed (*p* > 0.05) or to EDSS (*p* > 0.05; data not shown).

**Figure 1:**
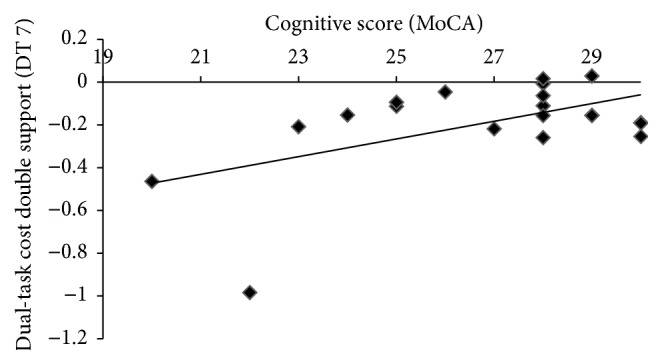
Dual-task cost of double support is related to cognition: in subjects with multiple sclerosis, lower cognitive score, measured by the Montreal Cognitive Assessment, is associated with greater dual-task cost of double support (*r* = 0.54,  *p* < 0.05). DT 7, serially subtracting 7's from 100, MoCA, Montreal Cognitive Assessment.

We found that neither EDSS nor SS walking correlated with DTC (*p* > 0.05) suggesting that people with greater physical impairment do not exhibit greater cost of DT. Importantly, we found that of the gait variables examined DTC of DS was the only variable correlated with cognitive score (*r* = 0.54, *p* < 0.05). MS subjects with lower cognitive scores (out of 30) had greater DTC of DS (Figure 1).

## 4. Discussion

Finding sensitive and reliable outcome measures that detect clinically important impairments in MS is paramount for future clinical rehabilitation trials. Dual-tasking is one such potential outcome measure. Our objective was to determine which DT method most consistently detected impairment in MS subjects and which gait variables were most important to consider. Although Leone and group have stated that “it still remains unknown which task has the most detrimental impact on DT performance” [7], we found that counting backwards by sevens (DT 7) produced the most consistent DTC in both MS subjects and controls. As suggested by Kalron and group, it is important that the tasks employed during DT testing are of adequate challenge in order to detect effects [16]. To our knowledge this is the first comparison of DT methods in MS subjects of varying disability and age, gender, and education matched control subjects. Our findings coincide with those of Muir et al. who showed that, in people with mild cognitive impairment and Alzheimer's disease, DT 7 while frustrating was effective in identifying DT impairments [18]. Although reciting alternate letters of the alphabet (DT ABC) has been examined in PwMS [10], we observed that the method was rhythmical, sometimes stabilizing rather than challenging walking.

We also sought to determine which gait parameter would be uniquely altered in MS subjects. Although other studies have focused on DTC of gait velocity [6, 10–12, 14–17, 22, 24], we found that velocity, stride length, and cadence were reduced in a similar manner among MS subjects and controls. Furthermore, the DTC of these variables were not altered by level of disability (EDSS score ≥ or <3) suggesting that DTC of velocity, stride length, and cadence are likely “blunt” tools to detect change as a result of intervention or the course of the disease. Although Sosnoff et al. reported differences in DTC of velocity between disability levels, the study divided subjects into three disability classifications and found differences only between the mild and severely affected groups [12]. Our comparison groups were more alike in disability level, suggesting that DTC of velocity did not detect smaller differences in EDSS scores. In terms of other gait variables, since other researchers have identified gait variability as a potential indicator of impairment in DT, we examined the variability of gait by calculating the coefficient of variability and found no effect of DT in any groups. Similarly, Hamilton and colleagues found no significant differences in DS variability between MS subjects and control groups [15].

Percentage time in DS, at SS pace, was a uniquely altered variable in some MS subjects, with greater DS time in high disability MS subjects compared to controls. We did not find a significant difference between the low disability group and controls. In contrast, Kalron and colleagues were able to distinguish patients with clinically isolated syndrome (CIS) from controls [16] which may have been related to the fact that subjects had recent onset of neurological symptoms (<90 days). We also show that DS increases significantly in MS subjects during DT but not in controls. Our findings conflict with those of Motl et al. who showed that DS was not significantly altered by DT in a sample of 82 people with RRMS [11]. Nogueira and colleagues also reported that DS increased during DT in both MS subjects and controls [24]. The discrepancies may be related to the fact that we tested subjects with greater variability in walking disability.

In our study, stride width and DTC of stride width emerged as unique indicators of MS-related impairment. As reported by others, we found that, at SS speed, as disability level increased, the base of support increased [12]. When collapsing the MS groups, we report a paradoxical phenomenon in which stride width is either narrowed or unaltered during DT in MS subjects and widened in control subjects which suggests a maladaptive balance response in MS subjects. Our findings concur with those of Nogueira et al. who reported that, in people with MS, step width (calculated in a similar way to stride width) decreases by about 1 cm during a dual task whereas it increases the same amount in controls [24]. Several authors have shown that stride/step width is not altered during DTC in MS; however previous studies have tested less severely affected subjects [12, 16]. Gunn et al. observed that 70% of MS patients experienced falls over a three-month period [13]. Older people who experienced lateral falls have narrower step width compared to other direction fallers [25]. Our findings suggest that stride width may be a reasonable measure of DTC; however this should be confirmed with a larger study cohort.

We also aimed to determine the relationship between DTC and cognitive and physical status. Confirming the work of others [9, 10, 14], we found that although walking at SS pace is related to physical disability (EDSS), the cost of DT walking is related to cognition. We found that only DTC of DS was significantly correlated with cognitive score (measured by MoCA) but not EDSS or walking at SS pace. In MS subjects, greater cognitive impairment is associated with greater increases in DS during DT. Contrary to our findings, others have reported correlations between DTC and EDSS scores [11, 14, 20]. The discrepancies may be related to the degree of cognitive challenge employed during the DT in these studies which were relatively simple word generation tasks or counting forwards or backwards.

Although our results provide new understanding of the use of DT to detect cognitive interference in MS, there are some limitations. We chose three tasks, all requiring good language ability. Future studies should evaluate other methods that employ a nonlanguage task such as a visual cue. We did not examine the cost of DT on the cognitive task. Understanding the DTC on cognitive performance is an area that requires further exploration. We tested a group of MS patients with a range of walking ability; however we cannot assume that these findings apply to all people with MS.

## 5. Conclusion

Our findings indicate that DT 7 (serially subtracting 7's from 100) is the most reliable cognitive task to detect DTC of the gait variables assessed. Increased percentage time in DS distinguished the high disability MS group from controls. Control subjects widened stride width when challenged by a DT whereas MS subjects (with subgroups collapsed) paradoxically did not. Finally, physical disability (EDSS) in MS subjects was related to gait parameters at SS pace, but DTC was related to cognitive performance (MoCA), indicating that challenges for many MS patients to optimally multitask may be dependent upon their cognitive, not physical, disability.

## Figures and Tables

**Table 1:** Demographic and clinical characteristics of the study population.

Characteristics	Low disability (*n* = 11)	High disability (*n* = 9)	Controls (*n* = 13)
Gender	7 females 4 males	6 females 3 males	8 females 5 males

Age (years)	42.64 ± 11.16	48.44 ± 8.55	45.92 ± 11.31

Post-secondary education (years)	3.36 ± 2.11	3.33 ± 1.58	3.62 ± 0.96

Years with MS	9.73 ± 5.82	13.67 ± 8.28	N/A

EDSS score	1.59 ± 1.02 Range (0–2.5)	4.39 ± 1.39 Range (3.0–6.5)	N/A

EDSS: Expanded Disease Severity Scale. Values mean ± SD.

**Table 2:** Gait variables during self-selected walking and DT conditions.

	Low disability				High disability				Controls			
	SS	DT ABC	DT 7	DT 3	SS	DT ABC	DT 7	DT 3	SS	DT ABC	DT 7	DT 3
Stride length (cm)	135.64 ± 14.07	121.64 ± 16.66^a^	116.55 ± 19.09^a^	121.40 ± 16.55^a^	123.35 ± 25.96	114.87 ± 25.27^a^	110.77 ± 27.14^a^	115.91 ± 27.69^ab^	135.32 ± 11.89	128.79 ± 12.54	126.28 ± 14.48^a^	127.71 ± 10.79
Stride width (cm)	8.00 ± 4.69	7.14 ± 5.52	8.12 ± 5.55	7.61 ± 5.22	11.52 ± 2.94	10.76 ± 3.16	11.31 ± 2.93	11.60 ± 3.07	8.42 ± 3.41	9.20 ± 3.12^b^	10.18 ± 3.08^a^	9.40 ± 2.97
Double support (%)	28.61 ± 2.80^*∗∗*^	32.78 ± 4.67^a^	34.71 ± 5.87^a^	32.19 ± 6.09	35.14 ± 9.78^*∗*^	38.05 ± 11.71^*∗*ab^	40.40 ± 12.97^a^	39.16 ± 11.84^*∗*a^	27.25 ± 2.57	29.26 ± 5.78	30.48 ± 8.84	29.86 ± 5.62
Velocity (cm/s)	118.27 ± 12.54	76.12 ± 25.24^a^	72.31 ± 26.28^a^	76.31 ± 17.08^a^	101.29 ± 34.28	77.66 ± 29.84^a^	80.36 ± 35.89^a^	78.16 ± 36.43^a^	117.37 ± 15.72	91.96 ± 23.24^a^	87.88 ± 27.39^a^	91.45 ± 27.27^a^
Cadence (steps/min)	104.04 ± 8.68	74.01 ± 19.63^a^	72.92 ± 17.96^a^	75.77 ± 13.35^a^	94.07 ± 22.94	79.03 ± 22.38^a^	81.07 ± 27.63^a^	77.03 ± 26.59^a^	103.48 ± 10.56	85.82 ± 20.90^a^	83.63 ± 22.42^a^	91.45 ± 20.80

^*∗*^Different from same condition in control group (*p* < 0.05); ^*∗∗*^different from same condition in high disability group (*p* < 0.05); ^a^different from self-selected walking in the same group (*p* < 0.05); ^b^different from DT 7 in the same group (*p* < 0.05); SS: self-selected walking; DT ABC: dual-task while reciting alternate letters of alphabet; DT 7: dual-task while serially subtracting 7's from 100; DT 3: dual-task while counting, leaving out multiples and numbers including 3; DTC: dual-task cost; values are mean ± standard deviation.

**Table 3:** Comparing dual-task cost (DTC) of three DT methods.

	MS			Controls		
	DT ABC	DT 7	DT 3	DT ABC	DT 7	DT 3
Stride length DTC	8.88 ± 7.45^b^	12.69 ± 9.71	8.74 ± 7.61^b^	4.38 ± 10.48	6.42 ± 10.00	5.12 ± 9.95
Stride width DTC	5.83 ± 26.32^*∗*^	−0.34 ± 26.33^*∗*^	6.90 ± 33.08^*∗*^	−19.18 ± 42.53	−32.87 ± 50.61	−24.97 ± 50.66
Double support DTC	−11.70 ± 13.27^b^	−18.89 ± 21.79	−11.89 ± 15.60	−6.93 ± 15.31	−10.86 ± 25.71	−9.35 ± 15.19
Velocity DTC	30.16 ± 20.12	32.18 ± 21.78	31.12 ± 16.04	20.66 ± 21.70	23.98 ± 26.58	20.33 ± 28.14
Cadence DTC	22.31 ± 18.49	23.48 ± 17.53	23.16 ± 14.66	17.41 ± 17.97	19.34 ± 21.12	11.36 ± 21.21

^*∗*^Different from control group (*p* < 0.05); ^b^different from DT 7 in the same group; DT ABC: dual-task while reciting alternate letters of alphabet; DT 7: dual-task while serially subtracting 7's from 100; DT 3: dual-task while counting, leaving out multiples and numbers including 3; DTC: dual-task cost; values are mean ± standard deviation.
